# Current Practices in Assessing Professionalism in United States and Canadian Allopathic Medical Students and Residents

**DOI:** 10.7759/cureus.1267

**Published:** 2017-05-22

**Authors:** Nandini Nittur, Jonathan Kibble

**Affiliations:** 1 Medical Education, University of Central Florida College of Medicine

**Keywords:** hidden curriculum, assessment, professionalism

## Abstract

Professionalism is a critically important competency that must be evaluated in medical trainees but is a complex construct that is hard to assess. A systematic review was undertaken to give insight into the current best practices for assessment of professionalism in medical trainees and to identify new research priorities in the field. A search was conducted on PubMed for behavioral assessments of medical students and residents among the United States and Canadian allopathic schools in the last 15 years. An initial search yielded 594 results, 28 of which met our inclusion criteria. Our analysis indicated that there are robust generic definitions of the major attributes of medical professionalism. The most commonly used assessment tools are survey instruments that use Likert scales tied to attributes of professionalism. While significant progress has been made in this field in recent years, several opportunities for system-wide improvement were identified that require further research. These include a paucity of information about assessment reliability, the need for rater training, a need to better define competency in professionalism according to learner level (preclinical, clerkship, resident etc.) and ways to remediate lapses in professionalism. Student acceptance of assessment of professionalism may be increased if assessment tools are shifted to better incorporate feedback. Tackling the impact of the hidden curriculum in which students may observe lapses in professionalism by faculty and other health care providers is another priority for further study.

## Introduction and background

Development and assessment of professionalism in medical students have been garnering more attention in academic medicine within the past 15 years. The importance of effectively assessing professionalism in medical students has been highlighted in several studies showing a failure to recognize and remediate professionalism in students and showing deficits in preclinical years was associated with poor clerkship performance during third and fourth years [1-4]. Recent studies also show close parallels in unprofessional behaviors identified in students and similar lapses shown by physicians as reported by the state medical board [3,5-6]. However, several factors described below continue to hinder standardization of a “best” practice to assess these traits in medical trainees.

### Defining medical professionalism

In 1986, the Liaison Committee on Medical Education (LCME) created a requirement for North American medical colleges to include professionalism and ethics into their curricula. A standardized set of definition criteria was never established, leaving it up to each school to develop their own method of implementing and assessing these values [7]. One of the first obstacles to arise was agreeing upon set criteria encompassing the values of medical professionalism. In 2000, Swick [8] proposed a definition founded in the sociologic view of the nature of a profession, taking into account the uniqueness of a physician’s work. He characterized medical professionalism throuideasals such as high moral character, possession of humanistic values, and individualizing responses to a society’s needs. The goal of this was to encourage the idea of medical professionalism as a “basis of medicine’s contract with society,” a belief system of shared ideals and values to ensure the deliverance of high-quality care to society [8-9]. Two years later, the American Board of Internal Medicine released a physician charter echoing these ideas, outlining the definition of professionalism into fundamental principles and professional responsibilities, such as commitment to the principles of patient welfare, patient autonomy, and social justice [10]. The commonalities between these proposed definitions have been adopted by a majority of institutions in developing and implementing longitudinal assessment strategies to follow learners. Yet obstacles remain in applying these abstract theories of professionalism to student behaviors and standardizing a “grading” system [5].

### Standardizing assessment strategies for each stage of medical education

The process of socialization from a didactic environment to a clerkship and finally resident years exposes the medical learner to different issues at each level. Therefore it seems inappropriate to assess all levels against one set of criteria [11]. In a recent survey, among students, residents, and faculty, it was evident that the definition of “professionalism” had different focuses among each level of training. All three groups agreed upon a few themes, namely: knowledge and technical skills, patient relationship (establishing trust and confidence), and character virtues [12]. Within these overarching ideas, each group aspired to different ideas, reflecting their differences in acquired experiences. Students focused on the fear of hurting a patient and the desire for mutual respect between superiors and themselves; residents described the need to be succinct, available, and adaptable, with a focus on peer-based rather than patient based duty; physicians focused on themes most closely resembling the charter, stressing maturity, resiliency, and the concept of duty to the patient [12]. This discrepancy highlights another difficulty towards the implementation and assessment of a standard professionalism curriculum: learners in medicine are going through different phases of identity formation and assessment strategies must take this into account when looking at which behaviors to appraise [11].

### The detrimental role of the hidden curriculum

The hidden curriculum can be defined as behaviors and attitudes conveyed implicitly (and sometimes even unintentionally) by educators and physicians, which have been shown to possess a powerful formative component on medical students and residents [1,5,11,13-15]. Unfortunately, there has been multitude of surveys which reveals that a majority of students during clerkship years have witnessed unprofessional behavior from physicians and faculty [16-20]. Among these behaviors were inappropriate behavior or language, inappropriately revealing patient information, or speaking negatively about other faculty members [19-20]. Messages picked up from occasionally observing bad behaviors from faculty undermine the teachings attempted by educators in didactic years. In fact, several assessment studies and literature reviews have shown a regression in professionalism and moral judgment when transitioning from didactic to clerkship years [2-3,7,21-22].

### Specific objectives

The review sought to answer the following questions: 1) Within the last 15 years, has there been a trend towards a gold standard of assessment in professionalism? 2) Has a standardized definition of “medical professionalism” been agreed upon? 3) What is the most commonly utilized assessment tool? 4) Have developmentally relevant approaches to the assessment of professionalism been described? 

## Review

A literature search was conducted on PubMed with the initial input query: ("medical professionalism" or"professionalism") and ("medical school" or"allopathic" or"medical student").

**Figure 1:**
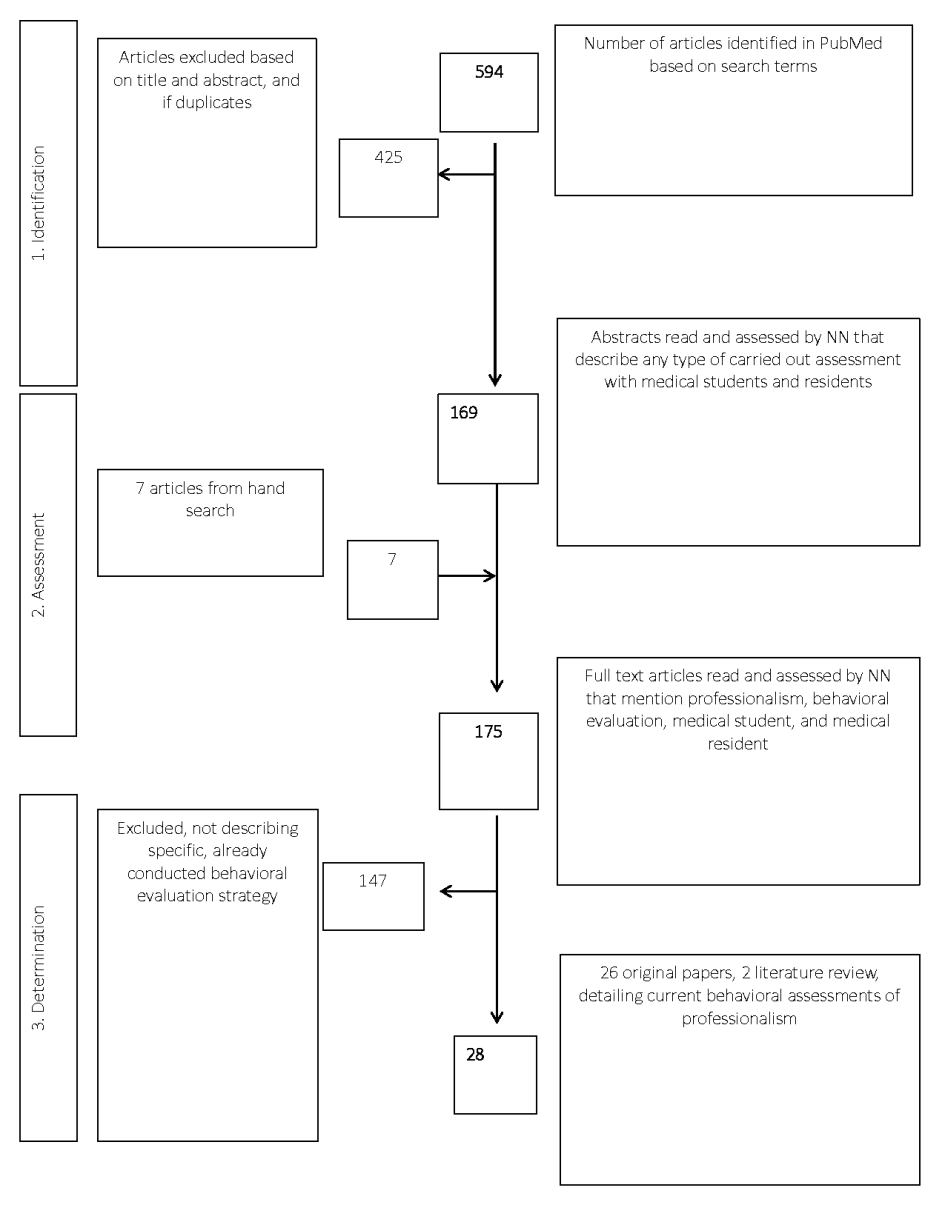
Flowchart showing phases of literature inclusion and exclusion

An initial review of results was based on the relevance of the abstract to the inclusion criteria. Articles that were included in the identification phase were recorded in a spreadsheet if any assessment strategy involving allopathic medical students or residents was described. During the assessment phase, a secondary review was conducted by reading the chosen papers and considered for inclusion if the study specifically described assessment strategies of student behavior that had already been conducted. As shown in Figure 1, a total of 28 articles fit the inclusion criteria and were used to make inferences about the trends of medical professionalism assessment at allopathic programs in the US and Canada.

Parameters captured include the type of study, location (U.S. vs. Canada), medical students or residents, situations where behavior was assessed, major lapse categories, described remediation strategies, formal training of assessors, assessment tool, longitudinal vs. crosswise, didactic versus clerkship years and any study limitations.

The articles chosen for inclusion in this review largely yielded descriptive details of assessment strategies in practice at US (n=24) and Canadian (n=3) allopathic schools. Out of 28 papers, three were literature reviews. The majority of papers (n=14) are listed as cohort studies, n=7 are observational, and n=4 are descriptive. A little over half (n=16) focused on medical students (both in didactic and clerkship years), n=5 described resident assessments, and n=3 were mixed (residents and students). Two of the three literature reviews were surveys of both U.S. and Canadian programs. Table 1 shows a detailed description of the papers meeting inclusion criteria that were reviewed in detail.

**Table 1:** Articles that met inclusion criteria

Title	Citation	Journal	Design	Location	Population	Sample
*Developing professionalism via multisource feedback in team-based learning.*	Emke, et al. (2015)	*Teach Learn Med*	Cohort	US	Students	0
*Validation of a performance assessment instrument in problem-based learning tutorials using two cohorts of medical students.*	Lee and Wimmers (2015)	*Adv Health Sci Educ Theory Pract*	Cohort	Canada	Students	310
*How do medical schools identify and remediate professionalism lapses in medical students? a study of U.S. and Canadian medical schools.*	Ziring, et al. (2015)	*Acad Med*	Literature review	US & Canada	Students	0
*Reflective practice: assessing its effectiveness to teach professionalism in a radiology residency.*	Kung, et al. (2015)	*Acad Radiol*	Case-based	US	Residents	30
*Development and validation of a questionnaire to evaluate medical students' and residents' responsibility in clinical settings.*	Asemani (2014)	*J Med Ethics Hist Med*	Cohort	US	Students, Residents	72 student, 69 resident, 32 intern, 64 extern
*Teaching big in Texas: team-based learning for professionalism education in medical schools.*	Lunstroth and Boisaubin (2014)	*Virtual Mentor*	Descriptive	US	Students	240
*Development and evaluation of standardized narrative cases depicting the general surgery professionalism milestones.*	Rawlings, et al. (2015)	*Acad Med*	Observational	US	Residents	16
*How to assess communication, professionalism, collaboration and the other intrinsic CanMEDS roles in orthopedic residents: use of an objective structured clinical examination (OSCE).*	Dwyer, et al. (2014)	Can J Surg	Cohort	Canada	Residents	25
*Early detection and evaluation of professionalism deficiencies in medical students: one school's approach*	Papadakis, et al. (2001)	*Acad Med*	Descriptive	US	Students	0
*Medical student professionalism: are we measuring the right behaviors? A comparison of professional lapses by students and physicians*	Ainsworth, Szauter (2006)	*Acad Med*	Cohort	US	Students	90
*Assessing professionalism: A review of the literature*	Lynch, et al. (2004)	*Med Teach*	Literature review	US	Students	0
*Professionalism deficiencies in a first quarter doctor-patient relationship course predict poor clinical performance in medical school*	Murden, et al. (2004)	*Acad Med*	Cohort	US	Students	42
*Can professionalism be measured? The development of a scale for use in the medical environment *	Arnold, et al. (1998)	*Acad Med*	Cohort	US	Students, Residents	565
*Can there be a single system for peer assessment of professionalism among medical students? A multi-institutional study*	Arnold, et al. (2007)	*Acad Med*	Cohort	US	Students	1661
*A strategy for the detection and evaluation of unprofessional behavior in medical students*	Papadakis, et al. (1999)	*Acad Med*	Descriptive	US	Students	24
*Professionalism in medical education: An institutional challenge *	Goldstein, et al. (2006)	*Acad Med*	Descriptive	US	Students, Residents	0
*Accounting for professionalism: an innovative point system to assess resident professionalism.*	Malakoff, et al. (2014)	*J Community Hosp Intern Med Perspect*	Observational	US	Residents	55
*Peer assessment among first-year medical students in anatomy.*	Spandorfer, et al. (2014)	*Anat Sci Educ*	Cohort	US	Students	267
*Evaluating medical student communication/professionalism skills from a patient's perspective*	Davis, et al. (2012)	*Front Neurol*	Observational	US	Students	165
*Use of simulated electronic mail (e-mail) to assess medical student knowledge, professionalism, and communication skills. *	Christner, et al. (2010)	*Acad Med*	Observational	US	Students	89
*Using standardized patients to assess professionalism: a generalizability study.*	Zanetti, et al. (2010)	*Teach Learn Med*	Observational	US	Students	20
*Comparative efficacy of group and individual feedback in gross anatomy for promoting medical student professionalism.*	Camp, et al. (2010)	*Anat Sci Educ.*	Observational	US	Students	49
*The multiple mini-interview for selection of international medical graduates into family medicine residency education.*	Hofmeister, et al. (2009)	*Med Educ *	Observational	Canada	Residents	71
*Professional boundaries: the perspective of the third year medical student in negotiating three boundary challenges.*	Gaufberg, et al. (2008)	*Teach Learn Med*	Cohort	US	Students	42
*Use of unstructured parent narratives to evaluate medical student competencies in communication and professionalism.*	Liu, et al. (2007)	*Ambul Pediatr*	Observational	US	Students	412
*Assessing professionalism in early medical education: experience with peer evaluation and self-evaluation in the gross anatomy course.*	Bryan, et al. (2005)	*Ann Acad Med*	Cohort	US	Students	213
*The nature of qualitative comments in evaluating professionalism. *	Frohna, Stern (2005)	*Med Educ *	Cohort	US	Students	153
*Measuring professionalism in a physiatry residency training program.*	DeLisa, et al. (2001)	*Am J Phys Med Rehabil*	Cohort	US	Residents	72

### Assessment setting

Literature reviews were categorized according to the most reported assessment setting, which was shown to be problem-based learning (PBL) settings, and “on doctoring courses” [23-24]. Behavioral assessments among medical students in their didactic years were generally found to be longitudinal group settings, such as PBL group interactions or gross anatomy courses (n=9). Peer assessment (n=4) was reported as an evaluation strategy among students only. Of the four papers detailing peer evaluations, three were during group anatomy course situation [25-27] and one performed as a generalized assessment of their peer’s behaviors [28]. Another common approach (n=6) included assessments in clinical scenarios among students in didactic years, such as standardized patient encounters, and “on doctoring” courses. Four papers assessed behavior exhibited during “on doctoring” courses, which are structured to teach students physical examination skills and introduce them to patient interactions [1,3,5,22]. The fourth paper in this category was an assessment strategy using standardized patient encounters [29]. Students in clerkship settings (n=3) were evaluated through patient and faculty interactions [22,30-33]. Other miscellaneous assessment situations found among students included responding to simulated patient emails and responding to scenario tapes [28-29,34-36]. Nine papers discussed resident assessments, three of which were mixed population (students and resident) studies. Assessment settings were varied among “snapshot” assessment strategies that were set in optional one time case-based workshops [36-38], general resident tasks [22,30,39-,40], mini medical interviews (MMI) [41] and objective structured clinical examinations (OSCE) [42].

### Assessment tools

Literature reviews were listed under most commonly reported assessment tool by paper which was Likert-evaluation forms and qualitative feedback [23-24]. Over half (n=18) of the included papers utilized a validated point scale system broken down into behavioral subcomponents, used among both medical students and residents [25-27,29,31-32-33,35-39,41-43]. Eleven of these assessments specified Likert-scale evaluations of subcategories of professional behavior which were totaled up into a summative numerical score. Another common strategy appeared was qualitative evaluation forms (n=5) which provided descriptive feedback to students [1-3,5,23]. Qualitative assessments were only seen to be used with medical students and not residents. Another assessment tool seen exclusively among medical students were reflective practices (n=2) [22,44]. Goldstein, et al. [22] are reflected twice under the mixed population column (once as qualitative feedback, once as reflective practice), as medical students were evaluated with reflective practices while residents were evaluated with clerkship evaluation forms. Among both populations, there were assessments based on single time workshops or case-based PBL’s with questionnaires that were administered afterward and subsequently graded (n=3) [28,30,45]. In one paper with medical students, this case-based workshop included scenario tapes that recorded student feedback with binary question responses [34]. One tool used exclusively by residents was “negative and positive point system,” where certain tasks or behaviors seen in generalized resident tasks were rewarded or punished with points that were totaled quarterly [40].

### Assessor training

Of the 28 papers chosen for inclusion, four mentioned the formal training of the assessors in performing behavioral evaluations. Lee and Wimners [43] describe a category where tutors assessed four domains of PBL performance (professionalism, use of information, problem-solving, and group process). These tutors were given a three hour training period during which they discussed the accurate use of the assessment tool and were closely monitored by experienced tutors. The longitudinal nature of the relationship between the tutors and students (across nine PBL blocks spanning two years) minimized common rater biases, such as observational inaccuracy. In a survey of 93 Liaison Committee on Medical Education (LCME) accredited allopathic programs across the US and Canada, 32 responded positively to having a formal program to prepare staff for assessing professional behavior. While not explicitly described, the responses noted most of the assessor training to be “optional and not robust” [23]. A paper by Zanetti, et al. [29] describes using standardized patients as assessors alongside MD raters, using a professionalism assessment based on the American Board of Internal Medicine criteria. The rater training consisted of viewing practice case tapes while intentionally not explaining the professionalism assessment and scale to minimize bias while rating. This standardized patients practiced assessments by pretending they were the patient in the taped encounter and rating the student accordingly [29]. In a study of professionalism exhibited by medical students applying for family medicine residency positions through an MMI process in Canada, Hofmeister [41] describes a panel of assessors as including community family doctors, family medicine residents from teaching programs, as well as a mix of human resource specialists, medical, and language educators. All assessors were given a formal and mandatory two-hour training session about the assessment tools and stations at which to practice formal assessments.

### Identifying standard assessment practices

Given the LCME regulations to define and report professionalism standards, there were less scholarly publications than anticipated of currently undertaken practices. This made it difficult to discern best practices and judge validation through the use of instruments across different institutions.

The most common strategy to assess professionalism in students depends on Likert-scale forms. We were surprised not to find more open-ended forms and tools using more rich qualitative description and feedback, particularly, since an influential early paper in the field by Papadakis, et al. (1999) paper [5] describes form-based qualitative incidence reporting, a description of the assessment tool, situations in which behaviors were assessed, and the remediation process. Papadakis, et al. detailed how they were able to bridge the assessment between didactic and clinical years. While most papers we reviewed since this time reported assessment settings and tools, there was little documentation of remediation strategies; only one additional original study described a course for remediation [40]. Our expectation was to see a trend towards similar form-based qualitative assessment approaches while conducting this review, especially another literature review reported many schools in the U.S. and Canada utilizing a variation of the Papadakis peer evaluation form [23]. Another less common strategy was to use questionnaires provided to students with simulated scenarios such as simulated patient emails or videos of theoretical situations and getting student feedback on how they would respond to that situation. Reflective practices and qualitative assessments were reported very infrequently.

One area for future work is reflected, in that there was no clear evidence of developmentally based evaluation strategies from the papers reviewed. This could be in part due to the limitations in assessment setting consistency among didactic, clerkship, and residency years. Of note, some of the settings utilized in student evaluations included “on doctoring” courses, which included professionalism under clinical skills and patient communication. This is an area to explore in terms of standardizing a bridged assessment between the first two and last two years of medical school. When evaluating students in similar roles, there is a greater likelihood of monitoring for deficits throughout the medical undergraduate years. There were a few resident-based assessments included in this review, but no consistency in the tools used for behavioral evaluation. Perhaps the emphasis on professionalism accountability is less in residency, as it becomes an assumed trait in this population. Another area for future development would be to evaluate major lapses seen in residents such as are there lapses seen that may be consistent with some of the reported unprofessional behaviors in the hidden curriculum? Tailoring an evaluation strategy to track and remediate professional deficits that are more likely to show up in a resident’s duties remains an area to be explored.

### Role of assessor training

As previously mentioned, the inherent nature of grading or assessing professionalism is evasive because it is often hard to describe the presence or absence of theoretical traits and values to clinical scenarios [46]. Additionally, many times the nature of the relationship of medical faculty and students is limited, and faculty is hesitant to make snap judgments on a student’s behavior from one observed instance of a lapse in professionalism [5]. Encouragingly, there are not many lapses among medical students being reported at most institutions, with the most common complaint being fairly benign lapses in responsibility (missing deadlines, unexcused absences, tardiness, etc.,) [23,25,34,38,40] but this raises the question as to whether students are actually consistently displaying ethical behavior, or whether faculty and peers are still uncomfortable giving negative feedback [21,23-24]. Of the 28 papers fitting inclusion criteria in this review, only four defined some sort of formal measure taken to train assessors in evaluating professionalism. Even amongst the four papers, only one reported mandatory training [29]. The assessor training was not focused on evaluating professionalism directly. Professionalism was listed as a subcategory within other factors being assessed, as in the case of preparing standardized patients to assess clinical encounters or faculty to observe residents within MMI interviews. This low incidence in reporting highlights another deficit on the way to creating a nationwide standard of assessment. Guidelines should be adjusted to enforce mandatory rate training as part of a gold standard in assessment practices. One of the key barriers to providing honest and standardized feedback to students is the hesitance of assessors to rate students poorly. Another danger is faculty members labeling any behavior they disapprove of as “unprofessionalism” [15]. As such, students cannot be remediated effectively. Educators generally enter the field of academic medicine to help students grow and succeed but may feel cast into a punitive role by feeling forced into potentially harming a student’s academic or personal growth by providing a negative evaluation. This reticence in feedback hinders the student from reaching developmental professionalism milestones and sets them up for more severe backlash as residents or practicing physicians. Creating mandatory standards of assessor training at different developmental levels (didactic years, vs clerkship years, vs residency training) would work towards establishing a nation-wide definition of professionalism at each level of learning as well as reassure assessors in their role for providing honest and effective feedback.

### Developmentally relevant practices, the hidden curriculum, and medical student “Professionalism fatigue”

While not explicitly explored in the papers fitting our inclusion criteria, the hidden curriculum became a prominent theme when exploring the developmental impact on attitudes towards professionalism made on students in their clerkship years. Several surveys of medical students in their clerkships have shown high incidences of observing unprofessional behaviors exhibited by role model physicians [14,17-18]. These deficits are often internalized by students and as they approach the end of their medical undergraduate career, they become desensitized to the discrepancies in what they observe and what they were taught in didactic training, as they transition from outsiders to members of the community they have been training to join [15,21-22,47-48]. Even more troubling is a recent study showing a lack of any kind of progression or evolution in moral reasoning and professionalism in students across all four years of medical undergraduate education. Many students entering residency positions are no more professionally competent than they were entering medical school [7]. What causes the stunted growth seen in this field of socialization?

One explanation is professionalism fatigue, a pushback reaction to the recent focus in the past two decades on attempting to teach and assess professionalism [49]. Students can recognize unprofessional behaviors exhibited by faculty and physicians and are therefore more critical of the attempts to be punished for their own lapses in professionalism [21]. In a recent survey of allopathic medical students, one-third responded they felt the current forms of professional education implemented as “patronizing and demeaning” [46]. Another survey revealed students are coming to think of professionalism as “adopting a certain persona,” in which they felt they were acting according to a prescribed code of conduct. They felt there was a difference between a “good” and a “professional” doctor [50]. Students are coming to view professionalism as an “external and imposed construct,” an act they can switch on or off in order to pass testing standards such as objective structured clinical examination (OSCE’s) or under the surveillance of an attending, rather than being encouraged to instill and demonstrate the morals constituting medical professionalism [22,46]. When professionalism assessments are presented as a numerical value, with positive or negative feedback provided based on a Likert-scale, an obvious tendency is to study to achieve a high score and creating the persona of professionalism [49]. Emphasis is not being placed on the importance of these core values that will have on clinical performance and patient safety once students enter clerkship years and even into their careers. Despite this, according to a recent survey of medical students, most are receptive to the role of character development in terms of ethics and professionalism in their curriculum [46]. While the temptation may be to provide the quantitative assessment to students in order to create a sense of standardization, the student response has shown this to be an ineffective means of evaluation. Giving student’s qualitative feedback may foster an environment more open to open discussion of the values that define medical professionalism and make negative feedback seem less punitive and more constructive. An additional source of rich qualitative feedback that medical schools could incorporate would be from other healthcare students as part of the recent initiative to include longitudinal interprofessional education in medical school.

## Conclusions

Our literature review concluded that there are some robust general descriptions of the core attributes of medical professionalism, but, more work could be done to elaborate on how these should be manifested in different levels of learner. Rather than the fundamental tenets of professionalism varying by learner level, it may be more productive to observe the manifestations of each trait by learner level. For example, a window to assess reliability and responsibility in a first year student may be things like timeliness, preparation and engagement in class, handing assignments in on time etc., whereas the focus would shift during clinical years to fulfilling patient care responsibilities such as contributing to rounds and assisting residents with efficiently completing their tasks. Similarly, assessing relationships and communication in the first year should leverage things like team learning situations, such as anatomy lab, to judge aspects such as sensitivity to the needs of others and respectful conflict resolution. In the clinical setting, there are additional opportunities such as establishing interprofessional rapport, establishing relationships with patients and maintaining appropriate boundaries. It seems that now would be the perfect time for a national consensus conference to help medical schools define manifestations of professionalism at each learner level, as well as meaningful strategies for remediation. It will be important to include learners in such an initiative as they have a more immediate perspective on what behaviors are feasible and unique insights into what should be expected from self, peers and faculty mentors.

While rubrics using Likert scales seem to be the most common tool for assessment, a greater educational impact of assessment may be realized by increasing student feedback through qualitative documentation and coaching. Documentation of rater training is poor and should be a standard guideline to improve the reliability of assessments. There is a need for more research and sharing of strategies for remediation of lapses in professionalism. Strenuous effort is needed to eliminate the incidence of poor role modeling by faculty and thereby minimize the negative impact of the hidden curriculum. A more open reciprocal evaluation of professionalism in the learning environment may also reduce the tendency for student professionalism fatigue and cynicism. To effectively tackle the adverse effects of hidden curriculum and give everyone in the learning environment accountability for the highest standards of professionalism, it is critical that senior administration in both medical schools and hospital affiliates demonstrate a vested interest. This could include the requirement of standardized rater training programs, attention to prompt feedback and reciprocal evaluation between medical professionals and learners. 
